# An Ultra-High Field Magnetic Resonance Spectroscopy Study of Post Exercise Lactate, Glutamate and Glutamine Change in the Human Brain

**DOI:** 10.3389/fphys.2015.00351

**Published:** 2015-12-17

**Authors:** Andrea Dennis, Adam G. Thomas, Nancy B. Rawlings, Jamie Near, Thomas E. Nichols, Stuart Clare, Heidi Johansen-Berg, Charlotte J. Stagg

**Affiliations:** ^1^Oxford Centre for Functional MRI of the Brain (FMRIB), Nuffield Department of Clinical Neurosciences, University of OxfordOxford, UK; ^2^Section on Functional Imaging Methods, National Institute of Mental Health, National Institutes of Health, Department of Health and Human ServicesBethesda, MD, USA; ^3^Douglas Mental Health University Institute and Department of Psychiatry, McGill UniversityMontreal, QC, Canada; ^4^Department of Statistics and Warwick Manufacturing Group, University of WarwickCoventry, UK; ^5^Physiological Neuroimaging Group, Oxford Centre for Human Brain Activity (OHBA), University of OxfordOxford, UK

**Keywords:** brain, lactate, glutamate, magnetic resonance spectroscopy, exercise

## Abstract

During strenuous exercise there is a progressive increase in lactate uptake and metabolism into the brain as workload and plasma lactate levels increase. Although it is now widely accepted that the brain can metabolize lactate, few studies have directly measured brain lactate following vigorous exercise. Here, we used ultra-high field magnetic resonance spectroscopy of the brain to obtain static measures of brain lactate, as well as brain glutamate and glutamine after vigorous exercise. The aims of our experiment were to (a) track the changes in brain lactate following recovery from exercise, and (b) to simultaneously measure the signals from brain glutamate and glutamine. The results of our experiment showed that vigorous exercise resulted in a significant increase in brain lactate. Furthermore, both glutamate and glutamine were successfully resolved, and as expected, although contrary to some previous reports, we did not observe any significant change in either amino acid after exercise. We did however observe a negative correlation between glutamate and a measure of fitness. These results support the hypothesis that peripherally derived lactate is taken up by the brain when available. Our data additionally highlight the potential of ultra-high field MRS as a non-invasive way of measuring multiple brain metabolite changes with exercise.

## Introduction

In the non-activated state, the brain's energy needs are met primarily by glucose. However, the brain has been shown to be capable of using substrates other than glucose when they are available to support activity, including lactate (Schurr et al., 1997; Bouzier-Sore et al., 2003; Overgaard et al., 2012; Schurr, 2014), pyruvate (Cruz et al., 2001; Sharma et al., 2003), and ketone bodies (Nybo et al., 2003; Chowdhury et al., 2014). During recovery from exercise, lactate is perhaps the most important of these additional substrates (Dalsgaard et al., 2002) as during high intensity exercise there is an increase in blood and brain lactate (Quistorff et al., 2008; van Hall et al., 2009). It has even been suggested that lactate may be preferred to glucose, possibly ‘sparing’ brain glucose metabolism during exercise (Quistorff et al., 2008; van Hall, 2010; Schurr, 2014).

There is, therefore, a great deal of interest in the metabolic fate of peripherally derived lactate within the human brain as evidence emerges as to its role as a putative neuronal energy source (Pellerin and Magistretti, 1994; Bouzier-Sore et al., 2003; van Hall et al., 2009) and as a signaling molecule in neuronal plasticity (Yang et al., 2014). Whilst persistently elevated brain lactate may indicate pathology, transient increases are normal consequences of the energetic processes involved in vigorous exercise and are now also believed to be complimentary to healthy brain processes (Schurr, 2008; Dienel, 2012) and brain recovery (Bouzat et al., 2014; Brooks and Martin, 2015; Glenn et al., 2015).

It is now widely accepted that on-going cerebral activity results in the generation of lactate in the brain, with lactate produced within the astrocytes fuelling neuronal activities (Fox et al., 1988; Schurr et al., 1988; Pellerin and Magistretti, 1994) probably via the astrocyte-neuron lactate shuttle (ANLS) (Magistretti and Pellerin, 1999; Pellerin and Magistretti, 2012). Here, however, we are primarily interested in the fate of peripherally derived lactate generated during exercise. Lactate is both taken up and produced in the brain by reversible reactions (Hertz et al., 2014). In one direction, pyruvate is reduced to lactate while NADH is oxidized to NAD+. In the other direction, lactate is oxidized to pyruvate, and NAD+ is reduced to NADH. In this direction, pyruvate is hypothesized to be either fully oxidized, producing 6CO_2_ and 6H_2_O, or partially oxidized, to allow for carbon atoms to participate in exchange reactions (Cerdán et al., 2006; Massucci et al., 2013) resulting in *de novo* synthesis of amino acid neurotransmitters, particularly glutamate (Hertz and Dienel, 2004). It is therefore possible for the pyruvate derived from lactate to be both oxidized to fuel neuronal activity and act as a metabolic intermediate in exchange reactions, such as those involved in glutamate recycling.

Glutamate is a non-essential amino acid and is the main excitatory neurotransmitter of the cerebral cortex. It does not cross the blood-brain barrier and is therefore synthesized within the brain from glucose and a variety of other precursors such as lactate (Hertz and Fillenz, 1999). *In vivo* studies have established that the glutamate-glutamine cycle between glutamatergic neurons and glia is a major metabolic flux, reflecting synaptic glutamate release (Shen and Rothman, 2002). The glutamate-glutamine cycling flux is directly coupled to neuroenergetics (Sibson et al., 1998) and thus they are both important cerebral metabolites. The ability to measure glutamate and glutamine therefore will greatly increase our understanding of brain metabolic processes and aid in our understanding of brain lactate metabolism pathways.

Although there have been a number of studies measuring brain lactate changes following exercise (Ide et al., 2000; Dalsgaard et al., 2004; van Hall et al., 2009; Volianitis et al., 2011) these often involve invasive measures of venous blood from the dominant jugular vein and technology restricted to examining metabolites only present in blood leaving the brain. Proton Magnetic Resonance Spectroscopy (H^1^ MRS) offers an attractive alternative to directly measuring cerebral blood measures as it is able to characterize brain tissues in terms of the relative concentration of many brain metabolites, including lactate, glutamate, and glutamine. 7 T ^1^H MRS is non-invasive, non-ionizing, and has been widely used for human studies. Alternative methods exist which are capable of measuring changes in brain metabolites, but they have significant limitations. Positron Emission Tomography (PET) is able to quantify neurochemicals and their binding, but relies on ionizing radiation. ^13^C MRS offers an alternative approach, where signal from ^13^C can be recorded, either via an infusion of ^13^C MRS or using hyperpolarization. However, although these techniques are perhaps the gold standard for this approach they involve both lengthy and expensive infusions or are still largely in development, neither of which would be appropriate to investigate the question here.

To date, only two studies have used ^1^H MRS to investigate changes in brain metabolites with exercise (Dalsgaard et al., 2004; Maddock et al., 2011). Whilst Dalsgaard and colleagues (Dalsgaard et al., 2004) were unable to resolve to lactate peak, using similar methods Maddock et al. (2011) used MRS at 1.5 T to examine the change in lactate and a combined measure of the glutamate and glutamine (Glx) concentration following vigorous exercise. The authors reported a sustained increase in brain lactate following exercise together with a transient increase in brain Glx, suggesting that at least some of the lactate is only partially oxidized. Although these results provide important direct evidence for brain metabolite changes post-exercise in humans, there are some limitations to the study. For example, at lower field strengths, detection of the lactate signal is difficult (Dalsgaard et al., 2004), and it is not possible to distinguish between glutamate and glutamine, making it impossible to know which is driving any observed changes in the composite measure Glx.

The aim of this study was two-fold: to replicate the experiment conducted by Maddock et al. (2011) in a study of exercise-induced brain lactate uptake and to further examine the change in brain glutamate in the presence of increased lactate as a proof of principle for *in vivo* post exercise multi-metabolite measurements. Here we used MRS at 7T to measure the relative levels of lactate, glutamate, and glutamine before and after exercise in 11 volunteers. We hypothesized that exercise would lead to global cerebral lactate uptake which would be fully metabolized during recovery. We expected to be able to resolve the signal from glutamine and glutamate but did not expect any significant changes in other measureable metabolites.

## Materials and methods

### Participants

Eleven healthy, untrained, adult volunteers [mean age 30.0 years (range 22–41), 4 male] participated. The study was conducted in accordance with the Declaration of Helsinki and was approved by the Central Office for Research Ethics Committee [Oxford REC B (10/H0605/48)]. All participants gave their written informed consent prior to participation.

### Experimental design

**Figure 1:**
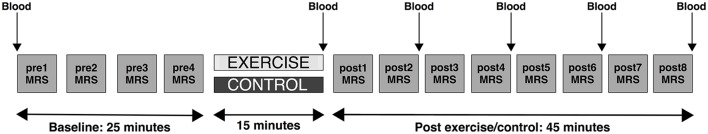
**Schematic depicting the study protocol**. Participants underwent two 90-min scanning sessions on two different days. On one of the days they received an exercise intervention. On the other day, they received the control intervention (quiet reading). The order of the days were randomly assigned and counterbalanced. In both sessions participants first underwent a series of baseline (*pre*) scans involving four repeated MRS measurements (depicted by gray squares), came out of the scanner for the intervention, and then returned to the scanner for a series of post-intervention scans (*post*) involving eight repeated MRS measures. Blood lactate measurements (denoted as “Blood”) were taken from the big toe before the pre-scan, at the end of the intervention period, and after post-MRS measurements 2, 4, 6, and 8.

Each subject underwent two experimental sessions: an exercise session and a control session. The order of the sessions was counterbalanced across the group. Sessions were conducted at the same time of day within subject. The experimental protocol for each session consisted of 20 min of baseline (*pre*) MRS measurements, after which subjects were removed from the scanner for the 15-min exercise or control intervention. They then returned to the scanner for 40 min of post-intervention (*post*) MRS measurement (Figure 1). During scanning, participants were instructed to lie awake but were not engaged in any task. Participants were asked to refrain from eating, drinking, or consuming caffeine in the 2 h prior to each session and to refrain from performing exhaustive exercise in the preceding 24 h.

In the exercise condition, subjects were required to perform a discontinuous, graded exercise paradigm to 85% of their predicted maximum heart rate (HR; HR_max_) (208–0.7 × age, ±3%) on a cycle ergometer (Monarch, UK). This protocol was selected to be comparable with the previous MRS/exercise study in the literature (Maddock et al., 2011), to allow for blood lactate to be rapidly produced endogenously without inducing severe fatigue that would make it difficult for participants to lie still in the MRI scanner, and without resulting in dehydration that might affect brain MRS measurements. Exercise began at 60 W for females and 90 W for males, and increased by 30 W every 3 min until 85% HR_max_ was reached. HR was measured using a Polar heart rate monitor (Polar Electro 20, Finland) and was recorded in the last 30 s of every 3 min stage. The mean length of this intervention was 14 ± 3 min. In the control condition, participants sat in a quiet seating area where they were provided with some light reading material for 15 min.

A proxy measure of aerobic fitness, the Physical Work Capacity (PWC), was established *post-hoc* for each individual in line with the PWC at 150 beats per minute test (PWC150; Campbell et al., 2001). Work rate achieved in the final step of the exercise protocol was normalized by body weight to give an estimate of PWC, measured in W kg^−1^ at 85% maximal heart rate.

In both sessions, blood lactate was measured from a capillary sample from the big toe before the *pre* scans, at the end of the intervention session, and every 10 min *post* intervention. Blood lactate was measured using the Lactate-Pro electrochemical test strip (Lactate Pro, Arkray Japan). The toe was used as a measurement site as access to participants' fingers was impossible when the participant was inside the 7T Magnetom scanner bore. Toe measurements were selected during exercise in order to match measurement site with those acquired in between MRS acquisitions. Measurements from the toe have been reported in the literature (Forsyth and Farrally, 2000; Garland and Atkinson, 2008) as a practical alternative site during exercise testing.

On reaching the end of the exercise test, participants were not afforded any time for active recovery. Continuing exercise at a lower intensity (active recovery) increases muscular blood flow (Bangsbo et al., 1994) facilitating the elimination of lactate. Instead, to maximize circulating lactate, subjects were returned to the scanner as soon as possible where they lay still for the duration of the post-exercise scans.

### Magnetic resonance data acquisition

Magnetic resonance (MR) data were acquired at the University of Oxford using a 7 T MRI scanner (Siemens Magnetom 7 T, Erlangen, Germany) with a 32-channel head coil (Nova Medical, MA, USA). Prior to MRS acquisition a high resolution T1-weighted sagittal structural scan [magnetization prepared rapid gradient echo (MPRAGE); Repetition time (TR) = 2200 ms; Echo time (TE) = 2.96 ms; Inversion time (TI) = 1050 ms; flip angle = 7°; voxel size = 0.7 mm isotropic; 256 sagittal slices; Field of View (FoV) = 224 mm] was acquired for anatomical overlay of MRS voxel location.

**Figure 2:**
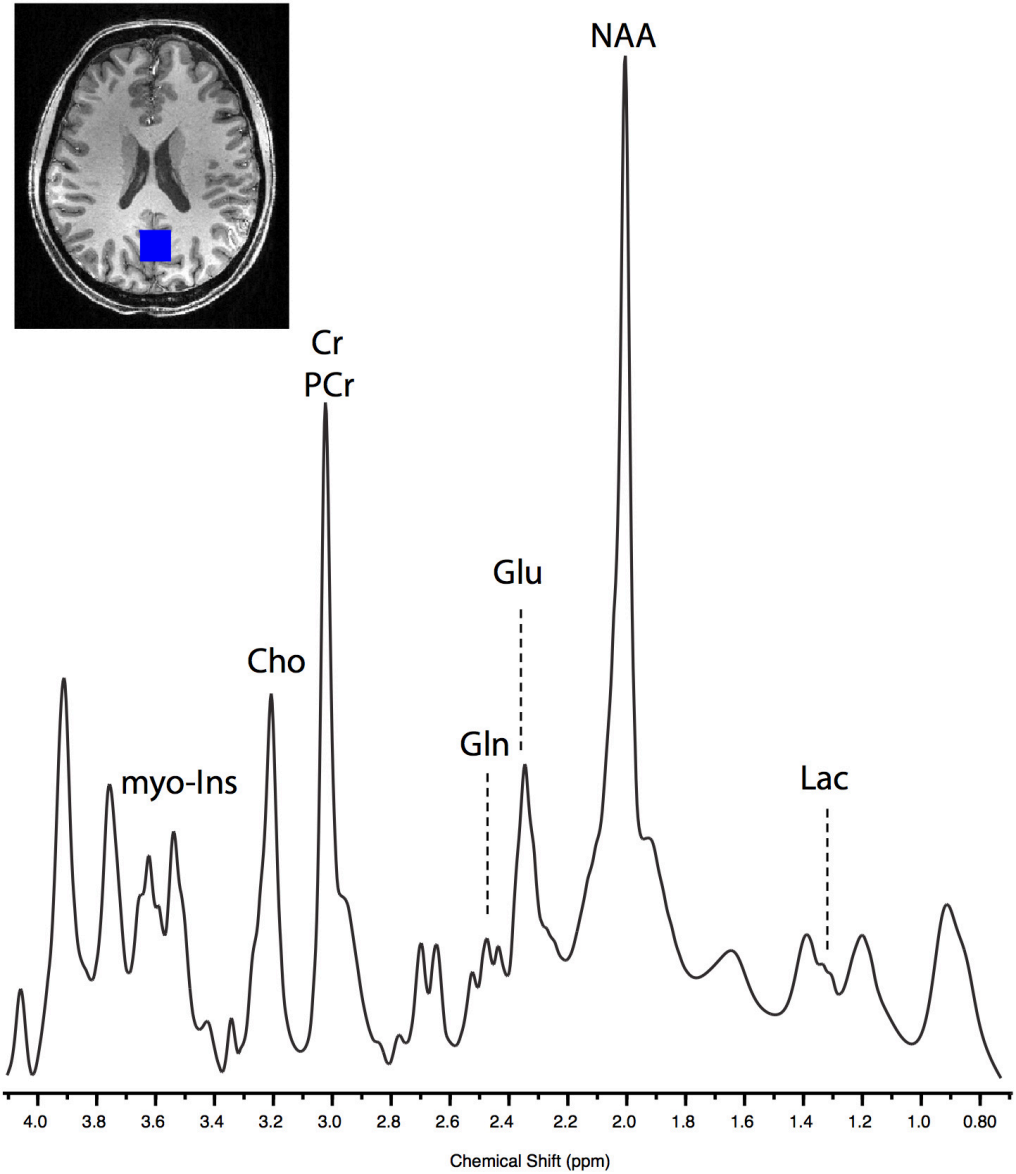
**Example of raw SPECIAL spectra acquired from one post-exercise block in a single subject**. The lactate peak can be seen centered at 1.33 ppm, and glutamate (Glu) and glutamine (Gln) can be clearly separated.

MRS data were acquired using an ultra-short TE spin echo acquisition, the spin-echo full-intensity acquired localized (SPECIAL) sequence (60 averages; TR = 4500 ms; TE = 8.50 ms) (Mekle et al., 2009; Xin et al., 2013). VAPOR (variable power RF pulses with optimized relaxation delays) water suppression was used (Tkác et al., 1999), and outer volume suppression was used to eliminate signal contamination from outside the MRS voxel. Shimming was performed using the Siemens product manual shim and the mean linewidth for the acquired data were 0.03 (*SD* = 0.006). An 8 cm^3^ (2 × 2 × 2 cm) voxel of interest was positioned in the occipital cortex (identified using anatomical landmarks). This voxel position was selected as it has been demonstrated to provide good quality MRS spectra and as any metabolite changes resulting from exercise would be expected to be global (Mekle et al., 2009). The voxel was centered on the mid-sagittal plane with its posterior boundary positioned 6 mm anterior to the skull at its point of closest proximity (Figure 2). Voxel placement was performed immediately after shimming on a rapidly acquired localiser scan prior to MRS measurement. Spectra were collected in blocks consisting of 60 acquired volumes, corresponding to a scan time of 4.5 min per block. Each scan session consisted of four *pre* time points and eight *post* time points. After the last MRS acquisition, a second T1-weighted structural scan was collected to allow registration of the *post* MRS voxels to those acquired at baseline.

### MR data processing

MRS data pre-processing was conducted in Matlab and included removal of motion corrupted averages (typically only one or two were removed), and retrospective frequency and phase drift correction by spectral registration (Near et al., 2014). Spectra were quantified using LCModel (Provencher, 2001). The basis set used was generated in-house using the FID-A Simulation Toolbox (http://github.com/CIC-methods/FID-A). Simulations consisted of an ideal spin echo sequence at 7 T with an echo time equal to that of our MRS acquisition (8.5 ms). Only metabolites that could be quantified with Cramér-Rao lower bounds < 25% were included in the analysis. The concentrations of all the metabolites were expressed relative to total creatine (tCr; creatine + phosphocreatine) as absolute quantification of metabolites has significant uncertainties, and we did not acquire simultaneous non-suppressed water spectra, so this approach may be misleading. However, in order to eliminate the chances of changes in metabolites being driven by change in creatine, statistics for absolute values are also reported.

To determine overlap between the *pre* and *post* voxels the two structural T1 scans were linearly registered using FLIRT (FMRIB's Linear Image Registration Tool; (Jenkinson and Smith, 2001) from the FMRIB software library (FSL; Smith et al., 2004). The *post* voxel masks were transformed into the space of the *pre* scan and overlap was determined using the fslmaths tool from FSL 5.1 (www.fmrib.ox.ac.uk/fsl).

### Statistical analysis

Statistical analysis was conducted on all *pre* and *post* measures of blood and of brain lactate, glutamate, and glutamine. To compare the effects of exercise vs. control, a longitudinal Linear Mixed Effects (LME) modeling approach was used, implemented in the *nlme* package of the R statistical programming language (Singer and Willett, 2003; Pinheiro et al., 2013). LME is similar to hierarchical linear regression in that explanatory variables are sequentially added and tested to determine if they explain a statistically significant amount of the variance to warrant inclusion in the final model, but has the advantage of separately modeling variance due to within and between subject factors (Singer and Willett, 2003). LME models have become a popular tool for analysing longitudinal neuroimaging data because of their flexibility to account for correlation within subjects (Bernal-Rusiel et al., 2013; Guillaume et al., 2014). Due to potential time-varying confounds, such as scanner drift, our null model was a linear change with time regardless of intervention.

All blood lactate values were log-transformed as blood lactate has a highly skewed distribution (Foster et al., 1978). To compare baseline levels of blood lactate between sessions, paired *t*-tests were performed. To explore the relationship between metabolite concentration and PWC, correlation analysis with a standard Fisher transformation was used.

## Results

Subjects' mean (±*SD*) weight was 68.0 kg (±13.7) and height was 1.72 m (±0.10). In the exercise condition, subjects exercised to an average of 87% (±3.6) of their age-predicted maximal heart rate. Average maximum exercise intensity was 185 W (±35). Average time post-intervention to the beginning of the first MRS acquisition was 32 min (±10).

Good registration of *pre* and *post* voxels was achieved for all subjects. The *pre* and *post* voxels within individual subjects overlapped in volume by 67.1% (± 4.7). Consistent with previous reports, LCModel resolved the spectra of 20 different metabolites reliably (Mekle et al., 2009).

### Exercise and brain creatine concentration

Although not initially a metabolite of interest in the study, the metabolic nature of the paradigm deemed it necessary to examine the effect of exercise on creatine as this was the metabolite by which all others are expressed in relation to. Total creatine was successfully resolved at 3.03 ppm, with successful measurement for all time-points. Using the LME approach; the model including the post-exercise variable (0 or 1) explained significantly more of the variance than the null model (likelihood ratio = 15.90, *P* = 0.0001) demonstrating that brain creatine was significantly decreased from baseline after exercise but not after the control intervention (Supplementary Figure 1). Based on this finding, all subsequent statistical analysis were conducted on both the metabolite/Cr ratio and on the absolute concentrations in order to rule out the effect of decreasing creatine on the observed patterns in lactate, glutamate, and glutamine.

### Exercise increases blood lactate concentration

**Figure 3:**
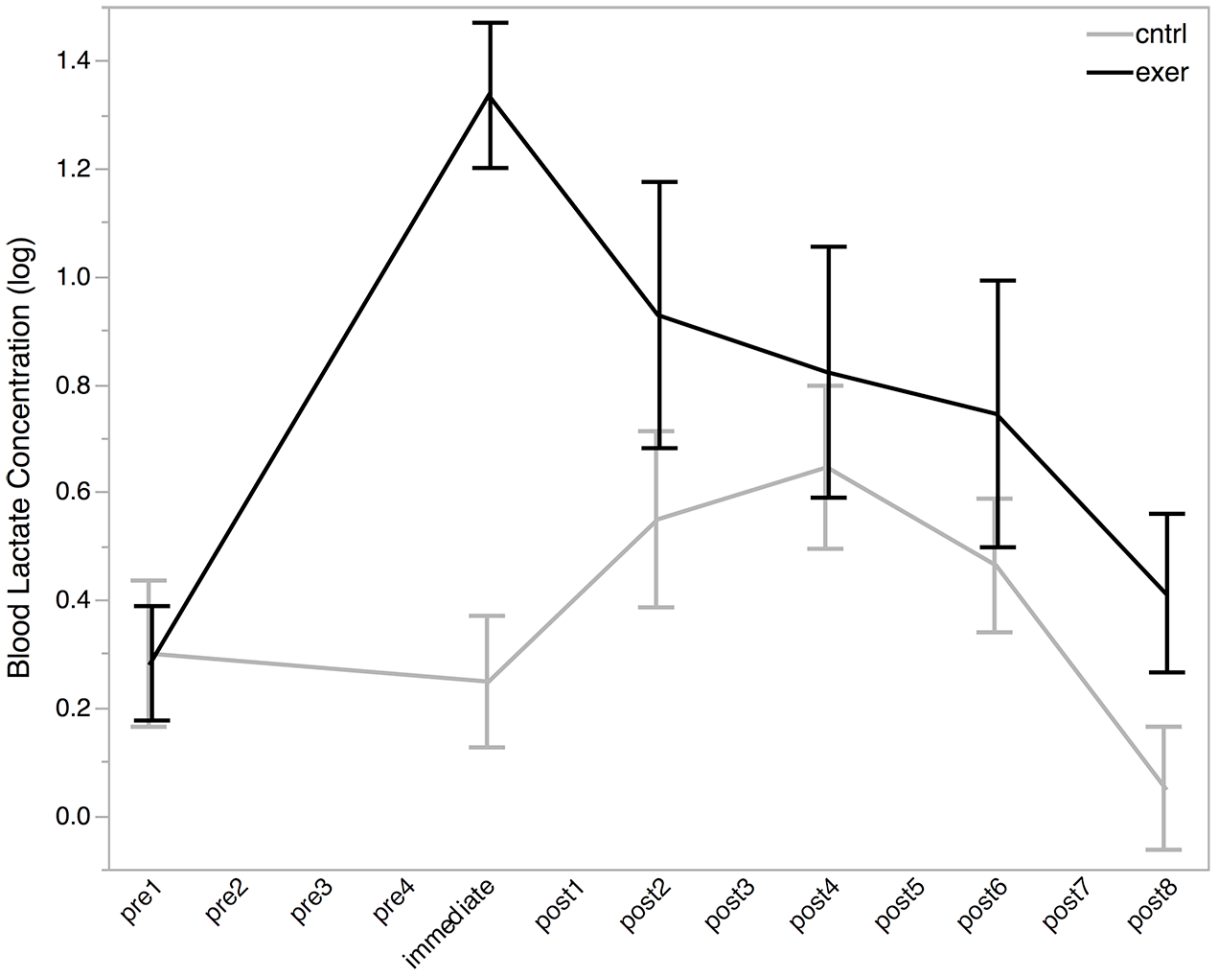
**Log blood lactate (log mmol/L) levels before (*pre*), immediately after, and *post* exercise (black line) and control (gray line) interventions**. Blood lactate measurements were not taken at each MRS measurement (see Figure 2). All MRS time points are shown on the x-axis for ease of comparison with MRS data. All data are plotted as mean ± standard error. There was no significant difference in blood lactate levels between the baseline conditions, but a significant effect of exercise was seen after the intervention (likelihood ratio = 30.176, *P* < 0.0001).

Blood lactate was acquired in 91% of cases. Mean blood lactate concentration was 1.4 ± 0.6 mmol/L at baseline. Paired *t*-test revealed no significant difference between baseline blood lactate levels in the two sessions [*t*_(10)_ = 0.12, *P* = 0.908]. Following the exercise session, blood lactate levels were increased from baseline to 4.2 ± 2.1 mmol/L (Figure 3). To test this change statistically, we used a LME approach. We constructed a model to explain changes in blood lactate across all subjects, sessions, and time points using three explanatory variables: subject ID, session, time, and a variable indicating whether the data point was collected after the exercise session (0 or 1). This model provided a significantly better fit for the variance in blood lactate than the null model which included only subject ID, session, and time (likelihood ratio = 30.176, *P* < 0.0001) demonstrating that exercise significantly increased blood lactate level. Importantly, this increase in blood lactate was maintained for at least the first two blocks of the *post* MRS measurements.

### Exercise increases brain lactate concentration

**Figure 4:**
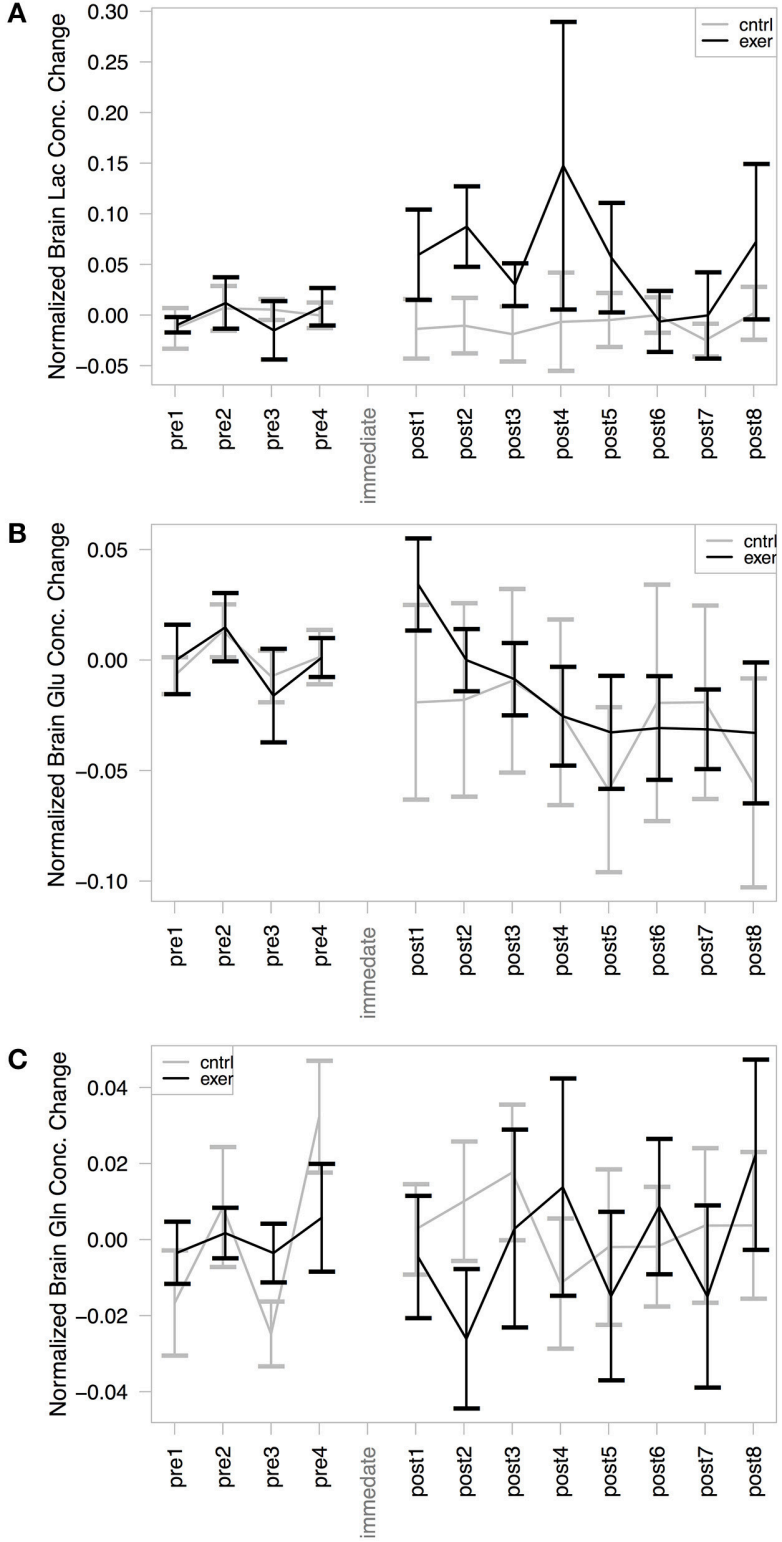
**MRS assessment of (A) Brain lactate/Cr; (B) Brain glutamate/Cr; and (C) Brain glutamine/Cr change from baseline within our voxel of interest**. In each case the exercise session is shown in black and the control in gray. The mean baseline concentration for each session is subtracted from the values to illustrate change. A significant effect of exercise was demonstrated in the post-exercise lactate levels, compared with those from the control session (likelihood ratio = 8.066, *P* = 0.005). No significant changes were observed in glutamate or glutamine concentration with either the exercise or the control condition.

As has been demonstrated previously (Mekle et al., 2009; Schaller et al., 2013) brain lactate could be accurately quantified at 1.33 ppm within the SPECIAL spectra (see Figure 2 for representative spectrum). Measurement of brain lactate was successful for 78% of MRS acquisitions. Using the same LME approach described above, we found that the model including the post-exercise variable (0 or 1) explained significantly more of the variance than the null model (ratio to Cr: likelihood ratio = 8.066, *P* = 0.005; absolute: likelihood ratio = 7.69, *P* = 0.006) demonstrating that brain lactate was significantly increased from baseline after exercise but not after the control intervention (Figure 4A). In a further analysis designed to assess the correlation between brain lactate and blood lactate, log blood lactate measures were added as a covariate into the LME model. Whilst this analysis did reduce the number of data points in the model (from 286 to 121) as blood lactate was measured after every other MRS acquisition after the intervention, this model provided a significantly better fit than the same model without log blood lactate included (ratio to Cr: Log likelihood = 3.839, *P* = 0.05; absolute: Log likelihood = 6.737, *P* = 0.0094). This indicates a correlation exists between blood and brain lactate measures.

### Glutamate and glutamine

Glutamate and glutamine were reliably resolved, with successful measurements at 99% of time points for glutamate and at 94% of time points for glutamine. There was no significant change in glutamate (Figure 4B) or glutamine (Figure 4C) in either session when normalized by creatine. The model including the exercise intervention was not a significantly better fit than the null model for glutamate (likelihood ratio = 1.158, *P* = 0.281) or glutamine (likelihood ratio = 1.759, *P* = 0.185), suggesting that exercise did not result in a significant change in either metabolite. However, when not normalizing by creatine, the models for both glutamate (likelihood ratio = 7.34, *P* = 0.007) and glutamine (likelihood ratio = 4.03, *P* = 0.045) showed a significantly better fit to the data when including the exercise variable.

### Effect of aerobic fitness

**Figure 5:**
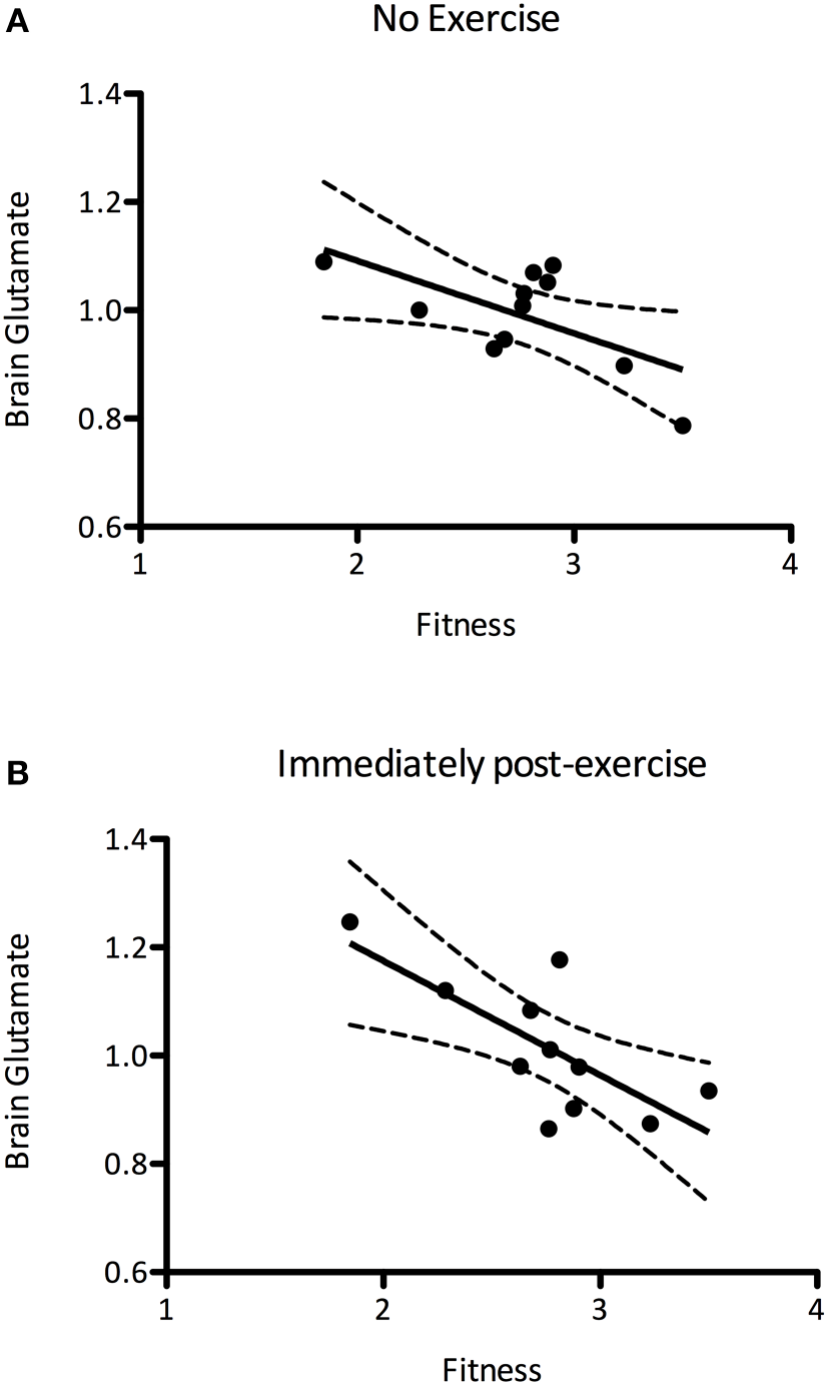
**(A)** A significant negative correlation between brain glutamate levels at rest and fitness (*r* = −0.627, *P* = 0.039), such that fitter people had lower resting glutamate levels. **(B)** This correlation is also present immediately after exercise (*r* = −0.726, *P* = 0.012). The relationship between brain glutamate levels and fitness are not significantly altered after exercise (Fisher's R-to-Z, *Z* = 0.12, *P* = 0.9).

It has previously been suggested that aerobic fitness modulates glutamatergic processing (Guezennec et al., 1998). In order to investigate this we first explored whether the mean baseline (*pre*) glutamate levels were related to the PWC150, a proxy measure of fitness. We demonstrated a significant negative correlation between baseline glutamate levels and fitness, such that fitter people had lower resting glutamate levels than less fit people (*r* = −0.627, *P* = 0.039, Figure 5A). We then went on to see whether this relationship could also be demonstrated immediately after exercise, i.e., whether any of the inter-subject variability in glutamate after the exercise condition could be explained by subject fitness. A correlational analysis revealed that brain glutamate immediately post-exercise and PWC were significantly negatively correlated (*r* = −0.726, *P* = 0.012; Figure 5B), such that fitter people had lower glutamate levels immediately post-exercise than less fit people. This relationship was not significantly different from that seen at baseline (Fisher's R-to-Z, *Z* = 0.12, *P* = 0.9).

## Discussion

This study investigated the effects of exercise-induced blood lactate increases on the levels of the brain metabolites lactate, glutamate, and glutamine in healthy adults. As in one previous study (Maddock et al., 2011), we have demonstrated a transient increase in brain lactate after vigorous exercise as measured through ultra-high field strength MRS. In the brain at rest, metabolite concentrations are likely to be homeostatically controlled (Balaban et al., 1986; Hochachka, 2003). Whilst significant increases in brain-derived lactate have been reported in healthy subjects during prolonged visual and motor stimulations (Prichard et al., 1991; Sappey-Marinier et al., 1992; Mangia et al., 2007; Schaller et al., 2014), such increases are transient and return to baseline within minutes. Therefore, there is no reason to expect an increase in brain tissue lactate between the *pre* and *post* scan in the current study, when neither acquisition required any task performance during data collection. We therefore believe our results support the hypothesis that lactate is taken up by the brain when available in high abundance in the plasma. However, unlike the previous study, we were able to resolve MRS peaks for both glutamine and glutamate. We did not observe any significant change in either amino acid following exercise, although we did observe a significant negative correlation between a proxy measure of aerobic fitness and glutamate.

The uptake and oxidization of lactate by the brain, heart, and muscle when available in the blood stream is not a newly discovered phenomenon, with a vast literature supporting its occurrence (Brooks, 1985, 2009; Schurr et al., 1997; Ide et al., 2000; van Hall et al., 2009; Bouzat et al., 2014). Here, for the first time, we present data from an ultra-high field MR study of brain metabolite concentration changes following exercise. This is an important advance, since MRS at 7 T allows for the accurate, non-invasive quantification of a wide range of metabolites, most pertinently here including lactate, glutamate, and glutamine. Although proton MRS cannot provide information on cerebral flux of metabolites between cells, or on cerebral compartmentalization, it can provide valuable *in vivo* information about brain metabolism.

Interestingly, we also observed a significant decrease in brain creatine following exercise. This is an important finding given the prevalence in the literature to ratio metabolites to the usually stable metabolite creatine. Further studies interested in the fate of brain metabolites following exercise should consider reporting absolute concentrations based on normalization to unsuppressed water spectrum.

### The metabolic fate of peripherally-derived brain lactate in the brain

A potential limitation with this type of experiment is that it is difficult to determine whether a change in lactate is due to increased brain uptake of plasma lactate or increases in locally produced lactate as a result of increased cerebral metabolism. However, results from our statistical analysis comparing brain lactate change were strengthened when blood lactate was added to the model, indicating a correlation exists between the brain and blood measures. This result, coupled with the absence of a change in glutamate and the temporal delay in acquiring our post-exercise measures, would be supportive of the increased uptake of plasma lactate. Thus, we believe our results support previous findings that lactate is taken up into the brain when it is in high supply in the bloodstream (Quistorff et al., 2008; van Hall et al., 2009). It has previously been shown that in the absence of exercise, all lactate taken up by the brain is oxidized, accounting for approximately 10% of cerebral energy requirements (Boumezbeur et al., 2010). During exercise, lactate accounts for 33% cerebral energy requirements (Overgaard et al., 2012), and may provide up to 66% of energy requirements when found in high concentration in the blood (Boumezbeur et al., 2010). Studies have suggested that the majority of plasma lactate is metabolized by the neurons (Boumezbeur et al., 2010). Whilst an interesting finding, and potentially in line with the hypothesis supporting a preferential use of lactate as a fuel source in exercising human subjects (Quistorff et al., 2008; van Hall, 2010), the MRS measurement is a total concentration and cannot dissociate between lactate within astrocytes and neurons; thus, we cannot conclude whether it is preferred to glucose following exercise. In addition, direct quantification of cerebral glucose levels was not possible with the MRS method used here and, therefore, we cannot comment on any changes in global brain glucose uptake.

In line with our hypothesis, but in contrast to a previous study in humans (Maddock et al., 2011), we did not observe any significant change in glutamate or glutamine following vigorous exercise, when the metabolites were normalized to creatine. When “absolute” quantification was performed, a significant decrease in both glutamate and glutamine after exercise was observed. As discussed earlier, presenting metabolites as a ratio to creatine is common practice as it is taken to provide a stable, simultaneously acquired reference metabolite, but here we see a significant decrease in creatine following exercise. In light of this, we also explored the changes in the absolute quantification of glutamate and glutamine, although we acknowledge there are uncertainties with this approach. With this analysis, a significant *decrease* in glutamate and glutamine was observed, in contrast to the increase observed by Maddock et al. (2011). It is not possible, therefore, given the discrepancy between these methods, to be confident as to whether there is a true decrease in glutamate and glutamine after exercise, but certainly our data do not support an *increase* in these metabolites, as previously observed.

Conceptually, glutamate concentration might be expected to increase in proportion to overall neuronal activity. During exercise, large global increases in cerebral metabolism are seen (van Praag, 2009). Evidence from the literature would suggest that non-oxidized carbohydrates, such as lactate, are only metabolized into glutamate when there is an increase in “brain work” demanding more glutamatergic neural activation, and an adequate source of ammonia (Hertz and Dienel, 2004). Therefore, measurements of glutamate, and lactate, taken *during* exercise may be expected to be elevated as a result of local production. However, our MRS measures were acquired some time after the cessation of exercise, and it is therefore unlikely that we would observe a heightened state of brain activity, and hence an increase in glutamate (MacIntosh et al., 2014).

Nevertheless, glutamate and glutamine changes may be observed in the presence of high brain lactate in some animal models (Waagepetersen et al., 1998; Hertz and Fillenz, 1999). For example, lactate may be partially oxidized in the TCA cycle allowing for glutamate to be oxidized to alpha-ketoglutarate, or for increased glutamate-glutamine cycling in the astrocytes in response to neural activity. However, these mechanisms largely depend on an increase in *brain-derived* lactate generated by the astrocytes in response to neural activation, and are not involved in response to an increase in plasma lactate. Studies of *peripherally derived* lactate uptake into the brain following either infusion of lactate or following exercise have reported virtually all (~87%) lactate taken up is recovered as CO_2_ (Volianitis et al., 2008; van Hall et al., 2009), suggesting that there is little carbon being exchanged from lactate to other metabolites (van Hall et al., 2009).

### Relationship between glutamate and fitness

Whilst previous studies have reported differences in the NAA/Cr ratio in frontal gray matter and Cho/Cr ratios in occipitoparietal gray matter between aerobically trained and sedentary adults (Gonzales et al., 2013) and a correlation between aerobic fitness and the frontal NAA/Cr ratio (Erickson et al., 2012), currently no studies have reported differences in Glu/Cr ratios between trained and sedentary individuals (Gonzales et al., 2013), potentially due to the methodological difficulties in separating glutamate from glutamine.

The negative relationship we observed between fitness and glutamate may provide some support to the beneficial effects of regular exercise participation on brain health. For example, in rat models, exercise training results in decreased glutamatergic activity in response to oxygen and glucose deprivation in hippocampal slices (Mourão et al., 2014), and lowers the glutamatergic input to the rostro ventrolateral medulla (Zha et al., 2013), a phenomenon associated with lowering blood pressure. Therefore, our finding of lower glutamate levels in fitter subjects could be related to the neuro-protective benefits of regular physical exercise. It should be noted however, that the exercise paradigm was not designed as an exercise test and thus a subsequent proxy measure of fitness (PWC) was inferred from the available data. In light of the interesting relationship between PWC and glutamate, an established test of aerobic fitness or lactate threshold may provide a more comprehensive metric for examining the relationship between fitness and brain activity. It should also be noted that, whilst an interesting trend, further study of a population with a range of aerobic fitness levels would be necessary in order to elaborate on any proposed relation between fitness and resting brain glutamate levels.

### Limitations

There are some limitations of the methods used in this study. The MRS technique allows for non-invasive measurement of neurochemicals *in vivo*, making it ideal for longitudinal studies in human populations. However, it has inherently lower signal-to-noise than more direct measures. MRS allows for measurement from a single ROI. The voxel in the visual cortex was selected based on the assumption that changes in brain lactate as taken up from the blood would be global and not regionally specific, and lactate peaks have previously been successfully resolved at 7 T in the visual cortex (Mangia et al., 2007; Mekle et al., 2009). However, this method required the voxel to be manually selected. Whilst every care was taken to ensure consistency, variations may have influenced the relative change in metabolites, though the reproducibility across the baseline and post-intervention time points suggests this is unlikely.

We only acquired an unsuppressed water spectrum at the beginning of the baseline scan and the post-intervention scan. We do not feel that this is an appropriate reference for the later post-exercise scans as hydration, as well as other physiological variables, might change. Secondly, there are significant uncertainties in calculating the “absolute” concentrations (highlighted in chapter 10 of the LCModel manual). Thirdly, we wished to extend the findings of Maddock et al. (2011), and so wished our results to be as comparable to that study, which used a creatine reference, as possible. However, given the discrepancy between our two quantification methods, future studies should include the appropriate scans to allow accurate absolute quantification to be performed.

It should also be acknowledged that the ROI approach gives us access to data from a specific region of the brain, which includes a number of constituents including brain tissue, CSF, and blood, and that in this voxel, with its proximity to the draining veins, the venous component might potentially be significant. However, we would argue strongly that the MRS measures we acquired here are not sensitive to signal from the blood within the voxel for a number of reasons. Whilst the initial excitation pulse will excite all the components of the ROI, the TE means that the vast majority of the blood that was in the voxel when the RF was applied will no longer be present, and therefore will not contribute to the signal acquired. This will be true of any arterial, arteriolar, and capillary supply within the voxel, where flow will be fast enough to remove a significant portion of the blood. Potentially, however, venous drainage in the sagittal sinus might be more sluggish than this. We were therefore careful to place the voxel sufficiently anterior of the sinuses to avoid this confound.

Whilst MRS offers an *in vivo* and non-invasive method for measuring brain metabolites, this “snapshot” measurement approach means only total, static measures are possible and measurement of flux is not. For information on metabolic flux, measures such as ^13^C MRS would need to be employed.

Lastly, the time-interval between the ending of the exercise session and the start of the first post-exercise scan was variable. It should be noted however that blood lactate levels were still elevated from baseline during first post-exercise MRS acquisition maintaining the integrity of the protocol to examine peripherally derived lactate. Further study of the changes in brain lactate and glutamate immediately after exercise may shed further light on brain metabolism and activity changes in the immediate recovery period.

## Conclusions

We observed a transient increase in brain lactate following vigorous exercise when blood lactate was elevated. We were also able to successfully measure brain glutamate and glutamine, and in line with our hypothesis, though contrary to previous findings (Maddock et al., 2011), we did not observe any change in either as a result of exercise. We did observe a negative correlation between brain glutamate and fitness, both after exercise and at rest. These results provide support for the use of high-field MRS for measuring post-exercise brain metabolites.

## Funding

The work was supported by the National Institute for Health Research (NIHR) Oxford Biomedical Research Center based at Oxford University Hospitals Trust, Oxford University. The views expressed are those of the author(s) and not necessarily those of the NHS, the NIHR, or the Department of Health. AT is supported by the NIMH Intramural Research Program based in Bethesda, MD. HJ is a Wellcome Trust Senior Research Fellow. CS holds a Sir Henry Dale Fellowship jointly funded by the Wellcome Trust and the Royal Society (Grant Number 102584/Z/13/Z).

### Conflict of interest statement

The authors declare that the research was conducted in the absence of any commercial or financial relationships that could be construed as a potential conflict of interest.

## Supplementary material

Supplementary Figure 1 **MRS assessment of absolute creatine (Cr + Pcr) within the voxel of interest**. Mean baseline concentration for each session is subtracted from the values to illustrate change. Exercise session is shown in black and the control in gray. A significant effect of exercise was demonstrated in the post-exercise creating levels, compared with those from the control session (likelihood ratio = 15.90, *P* = 0.0001).
