# Supplementary figures and images for: An Ultra-High Field Magnetic Resonance Spectroscopy Study of Post Exercise Lactate, Glutamate and Glutamine Change in the Human Brain
Source: Front Physiol. 2015 Dec 17;6:351. doi: 10.3389/fphys.2015.00351 (PMC4681779; doi:10.3389/fphys.2015.00351)

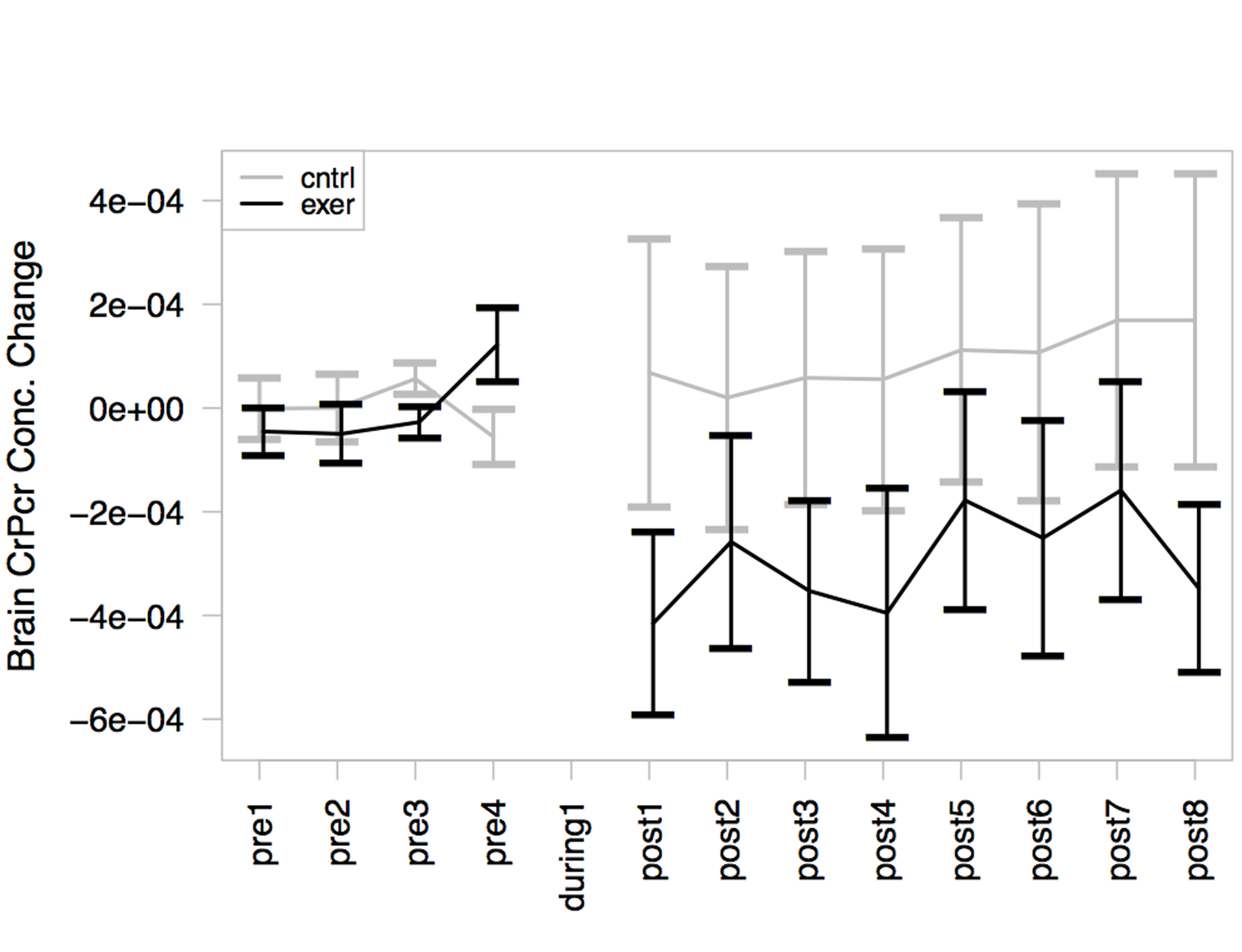

Supplement: Supplementary Figure 1 — MRS assessment of absolute creatine (Cr + Pcr) within the voxel of interest. Mean baseline concentration for each session is subtracted from the values to illustrate change. Exercise session is shown in black and the control in gray. A significant effect of exercise was demonstrated in the post-exercise creating levels, compared with those from the control session (likelihood ratio = 15.90, P = 0.0001). [file Image1.TIFF]
